# RGB-D-Based Method for Measuring the Angular Range of Hip and Knee Joints during Home Care Rehabilitation

**DOI:** 10.3390/s22010184

**Published:** 2021-12-28

**Authors:** Francesca Uccheddu, Rocco Furferi, Lapo Governi, Monica Carfagni

**Affiliations:** 1 Dipartimento Ingegneria Industriale, Università di Padova, Via Venezia 1, 35131 Padova, Italy; francesca.uccheddu@unipd.it; 2 Dipartimento Ingegneria Industriale, Università di Firenze, Via Santa Marta 3, 50100 Firenze, Italy; lapo.governi@unifi.it (L.G.); monica.carfagni@unifi.it (M.C.)

**Keywords:** RGB-D camera, 3D model, body tracking, joints estimation, data fusion

## Abstract

Home-based rehabilitation is becoming a gold standard for patient who have undergone knee arthroplasty or full knee replacement, as it helps healthcare costs to be minimized. Nevertheless, there is a chance of increasing adverse health effects in case of home care, primarily due to the patients’ lack of motivation and the doctors’ difficulty in carrying out rigorous supervision. The development of devices to assess the efficient recovery of the operated joint is highly valued both for the patient, who feels encouraged to perform the proper number of activities, and for the doctor, who can track him/her remotely. Accordingly, this paper introduces an interactive approach to angular range calculation of hip and knee joints based on the use of low-cost devices which can be operated at home. First, the patient’s body posture is estimated using a 2D acquisition method. Subsequently, the 3D posture is evaluated by using the depth information coming from an RGB-D sensor. Preliminary results show that the proposed method effectively overcomes many limitations by fusing the results obtained by the state-of-the-art robust 2D pose estimation algorithms with the 3D data of depth cameras by allowing the patient to be correctly tracked during rehabilitation exercises.

## 1. Introduction

The need for joint replacement surgery in the cases of end-stage arthritis is urgent in clinical practice today. Arthroplasty is, as is well known, an elective operation usually reserved for patients with persistent, debilitating symptoms that continue to occur amid exhaustion of all modalities of conservative and non-operative care. Over the next 30 years, literature studies predict a sharp rise in hip and knee replacements by older people who expect a longer and improved quality of life [1]. Rehabilitation after surgery is considered to be of utmost significance in preventing post-operative decline and maintaining a high degree of function [2]. The new trend for this kind of procedure is, in effect, to accelerate home discharge and allow patients to conduct a series of home recovery activities. However, with a shorter stay in the hospital, there is a chance of increasing adverse health effects, primarily due to the patients’ lack of energy and the doctors’ difficulty in carrying out tight supervision. Recently, several orthopedic medical centers implemented the so-called fast-track surgical protocol [3,4], which significantly enhances post-operative healing. Such a protocol allows decreasing hospital stay time from a median of five-six to three days, with reduced post-operative morbidity and no increased readmission rates. Fast-track procedures include early patient mobilization following anesthesia, early oral hydration and nutrition and removing any form of surgical treatment beyond 48 h, such as urinary catheterization or needle-tubes. Unfortunately, this method requires an exceedingly detailed control of the candidate’s whole perioperative time for complete knee/hip arthroplasty.

Therefore, patient knowledge is critical to carry out such a method; this can be accomplished by means of informative content and meeting with medical personnel. Starting from the pre-operative stage, the patient assumes an active part due to his awareness of his own healing measures. The task of an instrument for assessing the functional recovery of the operated joint is therefore important for both the patient to be encouraged to perform the necessary number of exercises and for the doctor/physiotherapist to be able to observe the patient remotely. To this end, it is crucial to measure the range of motion (ROM) of the knee/hip. Unfortunately, in this context, lateral radiography is widely recognized as the gold standard [5], but for normal post-operative complete knee/hip arthroplasty examination, radiographs of the knee/hip in both acute flexion and extension (or ab/adduction for the hip) are not indicated due to the risk of overexposing the patient to radiation.

Consequently, physicians prefer to assess a visual estimation, which is helpful but not accurate for the trained observer. In a number of cases, doctors simply use a goniometer or an inclinometer for measuring the angular range when the patient is within the hospital structure and do not have any control on the patient movements during exercises at home. Both instruments are poorly accurate for this kind of measurement, since a digital inclinometer appreciates a 6° minimum significant difference while the goniometer-based measurement could lead to even larger errors [5].

To address these issues, optical-based devices were exploited; not by chance, the most accurate approach to performing human body pose prediction is based on the use of marker-based motion capture systems.

**Figure 1:**
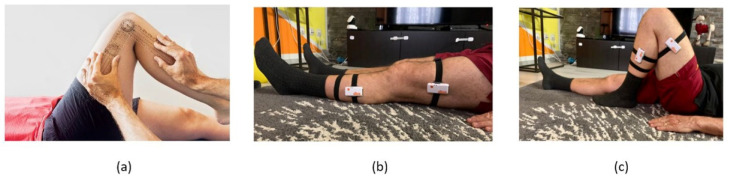
(**a**) A goniometer to measure the range of movements of a knee joint; (**b**) IMU sensors to detect the knee closure angle; (**c**) example of knee closure.

Not by chance, the use of optical-based motion capture techniques is therefore becoming a gold standard in healthcare environments to assess joint kinematics [6]. However, because such devices do not cause the subject to travel in his/her normal environment, the inertial measurement units (IMU) have given a local alternative (see Figure 1): 3D hip joint kinematics and 1D knee joint kinematics can be measured using a range of three IMUs.

Such instruments provide excellent results in terms of precision (i.e., less than 1 mm), but they are very pricey and require consumers to wear markers, thus restricting the technology’s large-scale diffusion, and more importantly, they constitute an obstacle to employment at home. The recent availability of smart cameras [7] and inexpensive RGB-depth sensors [8] enables cost-effective body pose evaluation and tracking. Unfortunately, while several low-cost, visual body- and hand-monitoring devices have been successfully used to measure the effect of post-operative exercises on the patient’s range of motion in the healthcare sense, the efficiency of the detector has remained very weak for non-frontal individuals [9]. Moreover, due to the greater 3D pose space, recovery of a 3D pose from 2D RGB images is deemed more complex than simple 2D pose estimation [10]. Motion capture systems or pose prediction algorithms from videos may obtain skeleton-based data. Finally, a variety of variables, including background scenes, illumination, garment shape and texture, skin color and image imperfections, among others, must be invariant in a reliable pose estimation algorithm [11,12].

To overcome the above limitations, the present work aims to provide a two-step interactive approach for the real-time monitoring of patients during their recovery treatment. The devised system addresses the key drawbacks of using a pure 2D or 3D skeleton tracking algorithm. In effect, comprising the use of both 2D and 3D acquisition devices, the method consists of a hybrid and interactive approach for the real-time measurement of lower limb joint rotation angles during post-total knee arthroplasty (TKA) and -total hip arthroplasty (THA) surgery recovery. First, a 2D acquisition method is used to estimate the patient’s 2D body posture. Then, the depth information coming from the RGB-D sensor is used to determine the spatial range of a patient’s lower joints so as to calculate the 3D positions of the joints. 

Consequently, it is possible to identify the joints of a human body in a complex environment in almost all the possible poses (i.e., including non-frontal poses) even though the output is purely the pixel positions of the joints. 

The developed method is able to measure the 3D locations of each lower joint (hip and knees) returned by the 2D tracker until the body posture is recovered by leveraging the depth information coming from an RGB-D sensor. Therefore, the angular extent of the patient’s lower joints may be calculated, and this detail may be used (by doctors) to ascertain the correctness of home-based therapy. The devised system relies on an experimental setup consisting of low-cost acquisition devices which can be installed within a home environment and can be operated by the patient after a short training.

## 2. Materials and Methods

**Figure 2:**
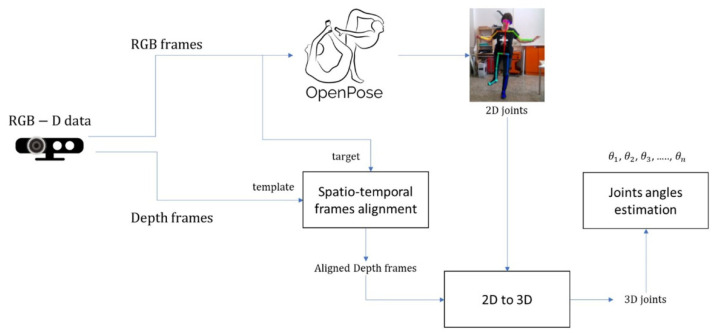
Overview of the proposed system.

The present paper proposes a new hybrid and interactive approach for performing a real-time measurement of lower limb joint rotation angles during post-TKA and -THA surgery recovery. The method exploits the details from a skeleton tracker added to an RGB video sequence as well as the 3D depth data of the same scene (through an RGB-D camera). A summary of the proposed method that begins from the patient’s acquisition of RGB-D data is in Figure 2. A depth camera (RGB-D) output is a double stream of RGB video frames and the corresponding depth video frames, the latter conveying frames with each pixel’s 3D coordinates depicted as a 2D gray-level image (i.e., the depth image). Three-dimensional data (depth frames) are used as a template for spatial-temporal alignment to obtain aligned depth maps [13]. Video frames from the RGB sensor are processed using the OpenPose library. As is widely known, OpenPose is a Caffe-based supervised convolutional neural network developed by Carnegie Mellon University (CMU) for real-time multi-person 2D pose estimation [14]. The posture estimation of human body gestures, facial expressions and finger movements may be learned. With an outstanding recognition effect and quick recognition speed, it is ideal for single- and multiple-user environments. Using part affinity fields and confidence maps, the OpenPose algorithm determines the human skeleton via a greedy algorithm to produce 2D skeletons for the characters.

**Figure 3:**
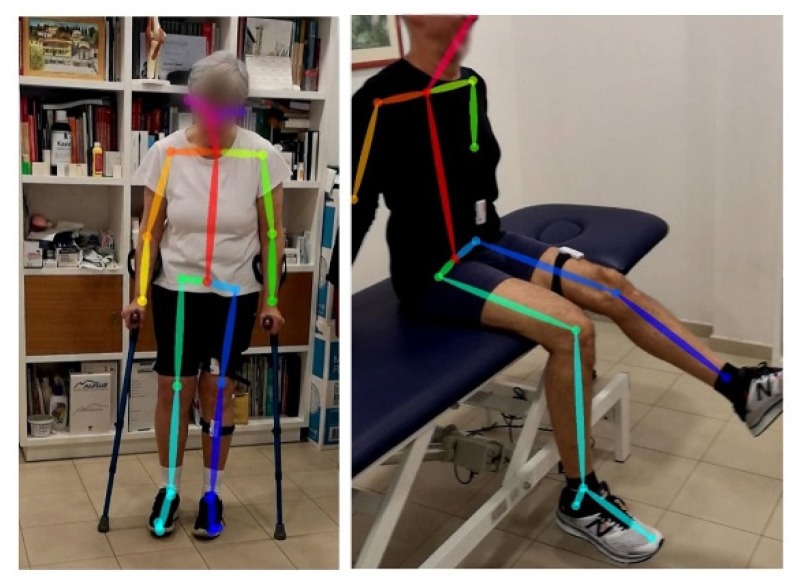
OpenPose video frame skeleton detection on a post-operative TKA (**left**) and THA (**right**).

Even for non-frontal poses, a robust 2D pose estimate can be achieved by using this method, as depicted in Figure 3. Each RGB frame will serve as a target for the above-mentioned depth frame alignment. The 3D positions of each joint are computed from the matched depth frames by leveraging the aligned depth information coming from the RGB-D sensor. The so-obtained 2D joint coordinates are used to infer a generic skeleton posture or to determine certain basic motions of the arms and legs. Nevertheless, the 2D joint coordinates are not adequate for most points of view to provide an accurate measure of knee and hip angles to this goal. Therefore, 3D joints coordinates are needed to provide hip and knee angles of flexo-extension as well as hip ab/adductor angles. Hence, the use of a depth camera allows for the retrieving of 3D pixel coordinates, allowing the angle of joints to be measured at all possible points of view for each motion.

**Figure 4:**
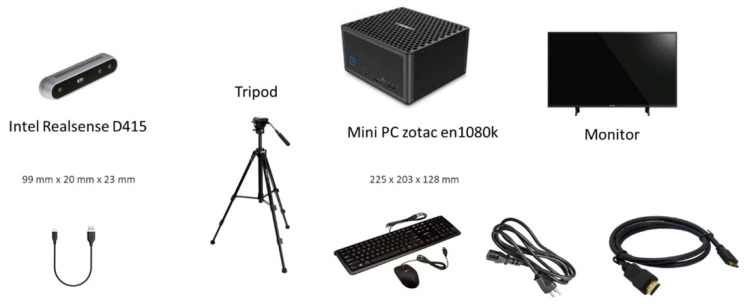
Experimental setup for home installation.

As shown in Figure 4, the above-mentioned technique requires the installation of an experimental setup, which can also be installed within a home environment. Such a system consists of a tripod with the Intel Realsense D415 depth camera (Intel, Santa Clara, CA, USA), a mini PC Zotac en1080k (Zotac, Hong Kong) with a GPU Nvidia GeForce GTX 1080 (NVIDIA Corporation, Santa Clara, CA, USA) and a standard monitor and cable connections.

The proposed method enables all the recommended movements to be tracked independently of the location of the standing or supine patient. To determine the position of the 2D skeleton joints from the RGB series, and to read the corresponding depth data from the depth video frames, the first step is to synchronize the two sequences both temporally and spatially. This is accomplished by using the Intel Realsense D400 series SDK [15], able to synchronize RGB and depth image acquisition and compensate for the offset between the two camera centers (i.e., RGB sensors for visible frames and NIR infrared sensors for depth frames).

**Figure 5:**
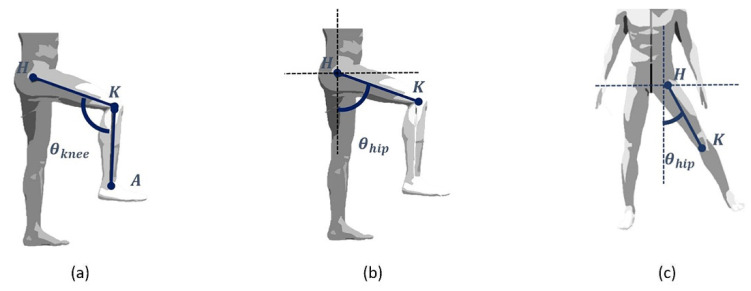
A simplified human joint/bones model of the lower limbs during (**a**) flex/extension of the knee, (**b**) flex/extension of the hip and (**c**) ab/adduction of the hip.

When the 3D space coordinates of the patient’s joints are established, it is important to measure the joint angles according to the particular activities the patient is doing to assess the achievable range of movements. In detail, the knee and the hip rotation angles can be measured, as depicted in Figure 5.

Since knee rotation is physiologically only possible on the plane defined by the two long bones, the knee rotation angle $\theta_{knee}$ can be assessed according to the following equation [16] to be evaluated during the flex/extension exercises:

(1) $$\cos(\theta_{knee})=\frac{<\vec{\left(H-K\right)}\;,\;\vec{\left(K-A\right)}>}{\left|\vec{\left(H-K\right)}\right|\;*\left|\vec{\left(K-A\right)}\right|}$$

where H, K and A are the 3D coordinates of the (left or right) hip, knee and ankle joints, respectively. Conversely, as the hip joint has more degrees of freedom, the calculation of the hip rotation angle $\theta_{hip}$ is more complicated.

**Figure 6:**
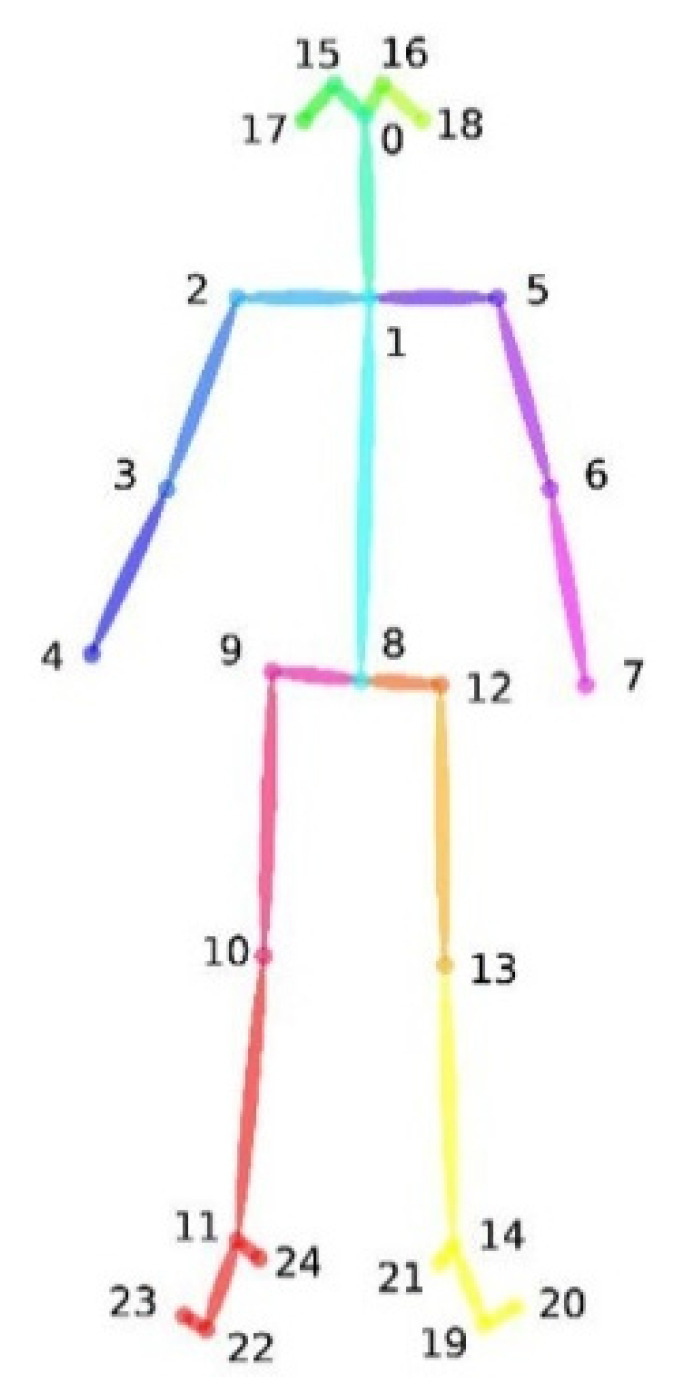
OpenPose Body-25 configuration of the human skeleton.

For this purpose, for each exercise, it is important to define a plane on which to project the hip and knee joints and to measure the angle of hip rotation. As shown in Figure 6, there are 25 joint points in the human body estimated by OpenPose: the nose, the right and left eyes and ears, the neck, the right and left shoulders, elbows and wrists, the center of the hip, the right and left hips, knees and ankles and the left and right soles, toes and heels.

The hip plane (i.e., frontal plane) is evaluated in real time as the plane passing through the neck joint and the right and left hip joints. Therefore, the flex/extension hip angle $\theta_{hip_{flexextension}}$ is evaluated by projecting the joints on the sagittal plane, according to the following equation:

(2) $$\tan\left(\theta_{hip_{flexextension}}\right)=-\frac{x_{H}-x_{K}}{y_{H}-y_{K}}$$

where $\left(x_{H},y_{H}\right)$ and $\left(x_{K},y_{K}\right)$ are the projection of the 3D coordinates of the right or left hip and the knee on the hip plane, respectively.

Finally, the hip ab/adductor angles are evaluated by using the following equation:

(3) $$\tan\left(\theta_{hip_{ab\_adductor}}\right)=-\frac{x_{H}-x_{K}}{y_{H}-y_{K}}\;$$

where $\left(x_{H},y_{H}\right)$ and $\left(x_{K},y_{K}\right)$ are the projection of the 3D coordinates of the right or left hip and the knee on the plane of the exercise that is perpendicular to the hip plane, respectively.

## 3. Results

**Figure 7:**
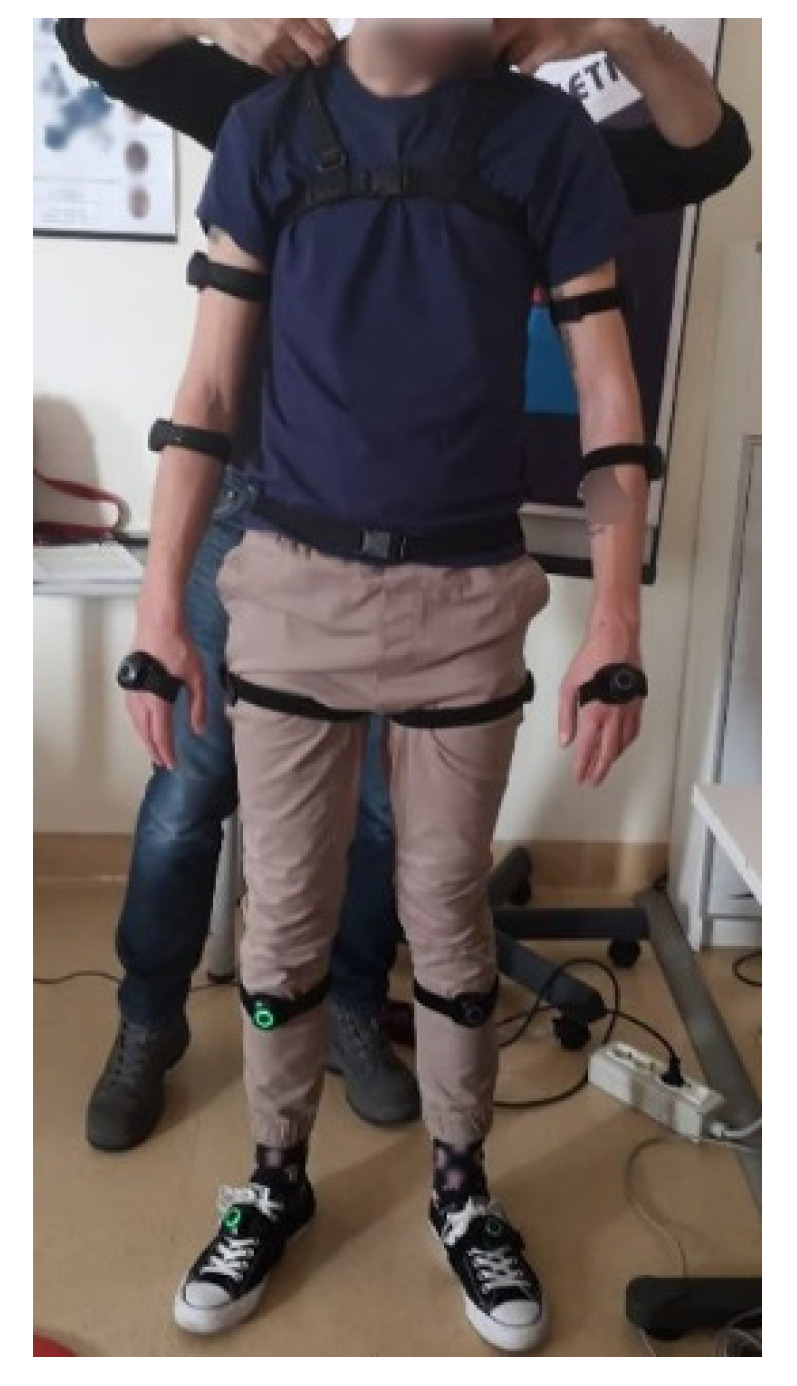
Physiotherapist wearing the motion capture device.

**Figure 8:**
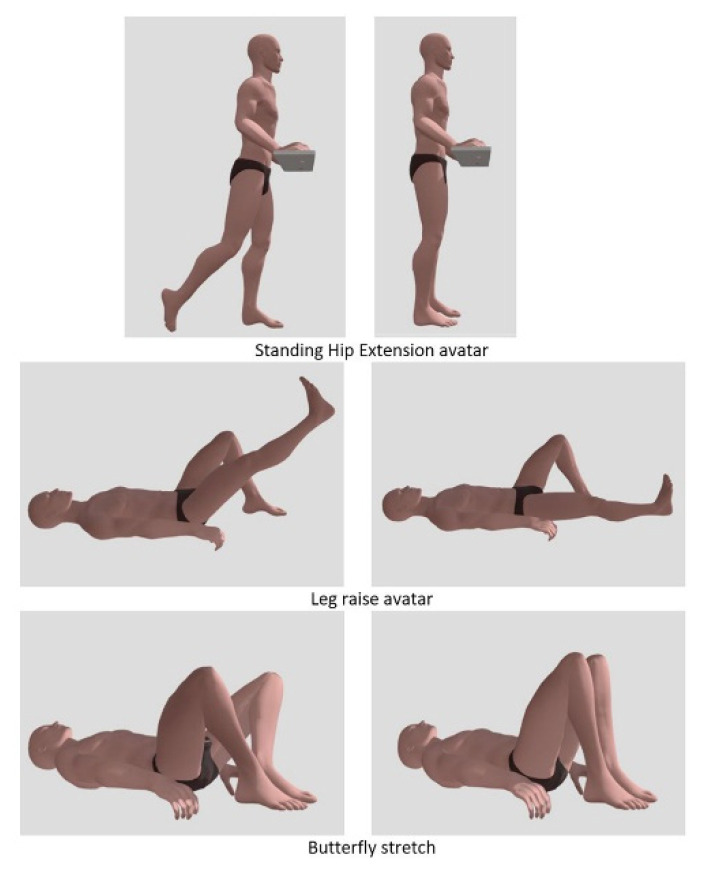
Example of three exercises recorded according the movements of a skilled physiotherapist and transferred to an avatar.

The performance of the proposed system was preliminarily validated through a testing phase. A trained physiotherapist (see Figure 7), who was filmed using a professional motion capture device to create a synthetic, rotatable and zoomable 3D video of an avatar (see Figure 8), conducted a preliminary test. Under the supervision of the authors, the tester was asked to carry out a series of different exercises to simulate the typical movements needed for a proper rehabilitation: standing hip extension, leg raise while lying down and butterfly stretch. The acquisition system was positioned to acquire an almost full image of the collaborator poses, while minimizing blind spots. Accordingly, the Noitom Perception Neuron IMU suit (Noitom, FL, USA) [17] was able to retrieve the full-body motion of the physiotherapist. In fact, this system provides the ability to perform calibrated full-body inertial motion capture in real time while streaming and logging kinematic data into their proprietary software (Axis Neuron). A three-dimensional reconstruction of the suit’s wearer is retrieved within the system’s proprietary software and, once optimized, consistent wearer movement can be visualized for all body segments. The suit has several operating modes, which include single-arm, upper-body and full-body capture. Each mode can utilize a different number of neurons (IMUs), ranging from three in single-arm mode to 32 in full-body mode. Within this study, the system was configured in the full-body, 18-neuron mode. Each neuron is an IMU consisting of a three-axis gyroscope, a three-axis magnetometer and a three-axis accelerometer. The recorded movements were transferred to an avatar and exported as an FBX file thereafter.

An appropriate single point of view was selected for the whole data set. The exercises mentioned in Table 1 and Table 2 were included in the doctor’s prescription for the first 7 days of remote physiotherapy after TKA and THA. 

**Figure 9:**
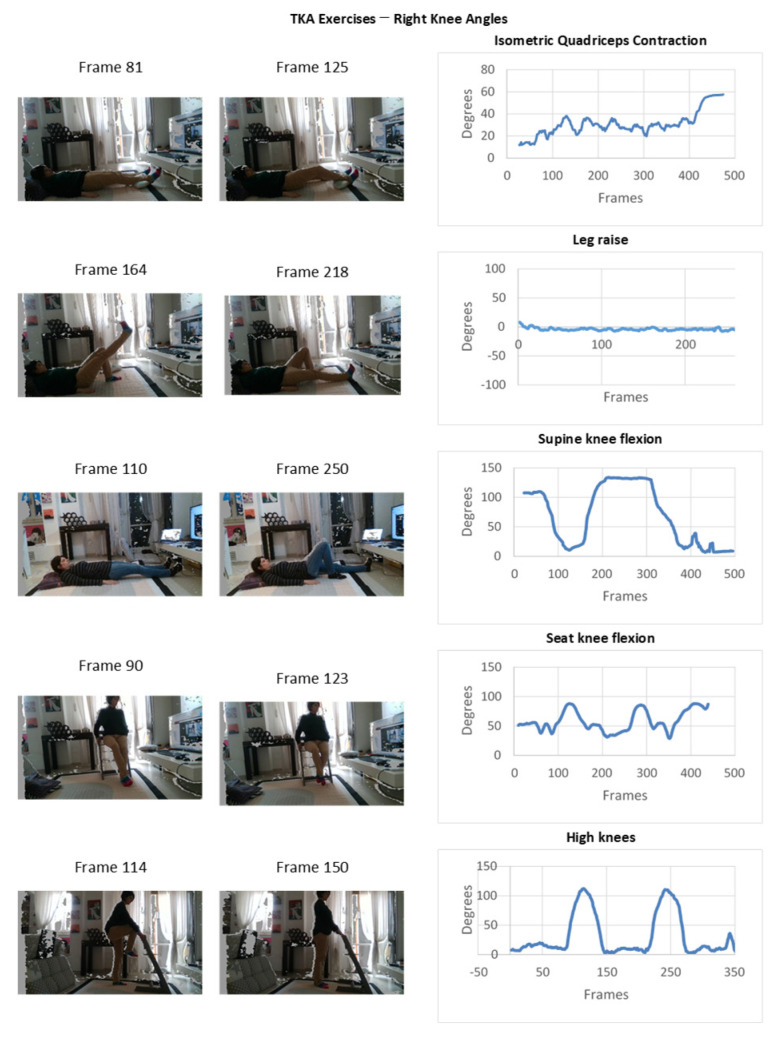
Experimental results of the monitoring in a domestic TKA post-operative environment.

**Figure 10:**
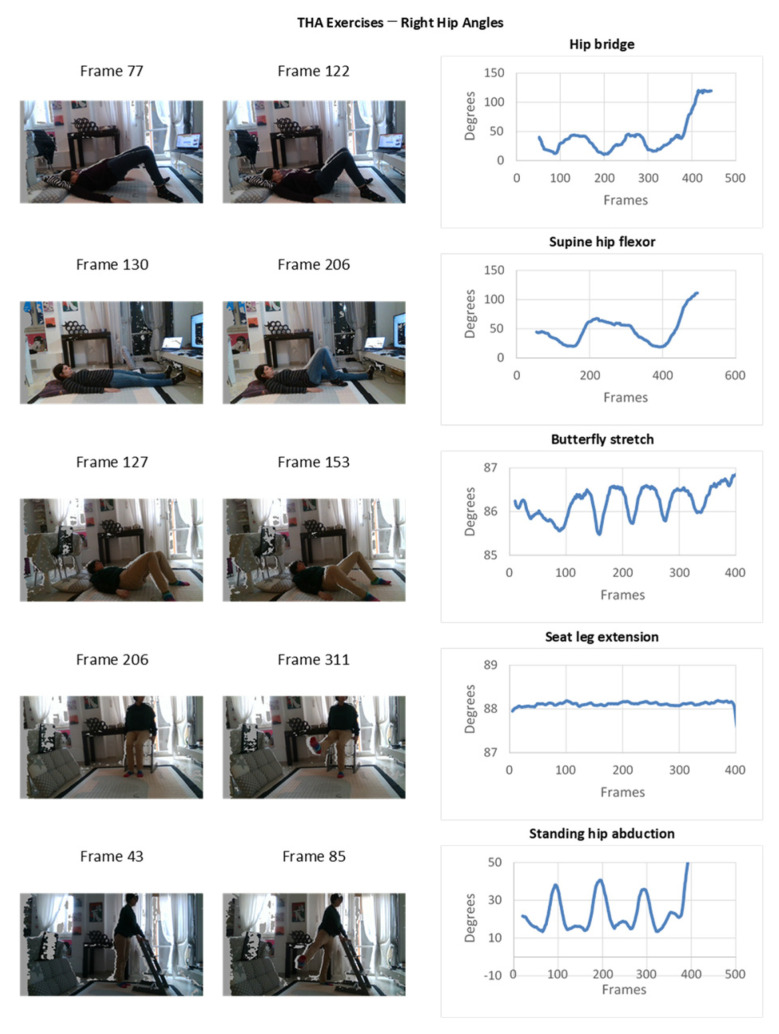
Experimental results of the monitoring in a domestic THA post-operative environment.

Experimental results of the monitoring in a domestic TKA post-operative environment are depicted in Figure 9 and Figure 10 with reference of the given frames of the 3D acquisition.

The obtained results can be preliminarily compared with the ones retrieved using manual methods, such as, for instance, the ones based on the use of a goniometer or the visual evaluation of the joints excursion angles during the execution of rehabilitation exercises. In the present work, a comparison is carried out with reference to the 3D avatar movement, which acts as ground truth of the testing procedure.

In particular, for a given acquisition frame, the desired angle can be measured in the 3D avatar within a CAD environment by using standard CAD-based measurement tools in order to compare such measurement with the one provided by the proposed automatic system. In fact, CAD measurement can be considered sufficiently accurate to be used as ground truth for the measurement [18].

**Figure 11:**
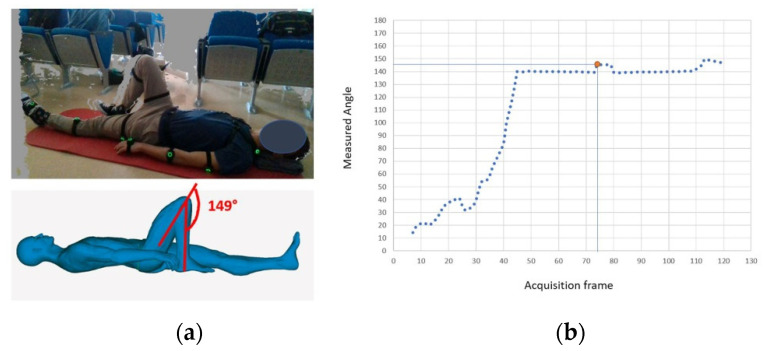
Example of the comparison between the (**a**) CAD-based measurement on the 3D avatar (ground truth) and the (**b**) automatic measurement for full contraction of the knee.

With reference to the retrieval of the prone hip flexor, as shown in Figure 11a, the selected frame is the one in which the patient fully contracts the right knee (frame 74). As already stated, the 3D avatar replicates patient movement, and therefore, it is straightforward to measure the tibiofemoral angle on it, which is, for the example in Figure 11, equal to 149°. Such a value can be compared with the one obtained with the automated system extracted for the selected frame (see Figure 11b) that equals 147.54°.

**Figure 12:**
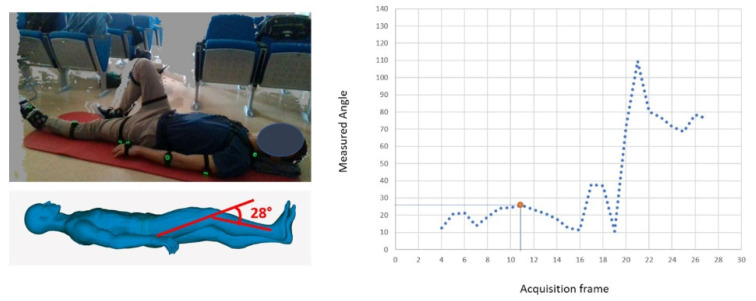
Comparison between the CAD-based measurement on the 3D avatar (ground truth) and the automatic measurement in case of isometric quadriceps contraction of the right knee.

**Figure 13:**
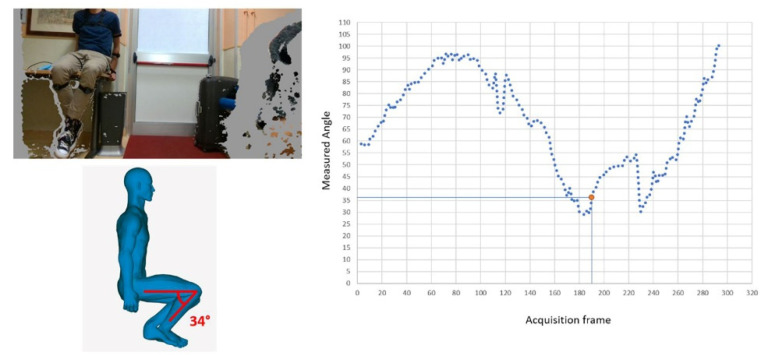
Comparison between the CAD-based measurement on the 3D avatar (ground truth) and the automatic measurement in case of seated knee flection.

Similar results can be obtained for different exercises, as demonstrated in Figure 12 (isometric quadriceps contraction of the right knee) and in Figure 13 (seated knee flexion, measured on the right knee).

For testing the system, the physiotherapist performed the measurements 45 times. The measurement of interest angles was performed for the set of 45 repetitions and compared to the CAD-based one. The absolute value of the error in identifying the prone hip flexor was 1.05°, with a variance of 0.4°. In the worst case, the measurement error was found equal to 2.23°. Referring to the isometric quadriceps contraction of the right knee, an average absolute error equal to 1.32° with a variance equal to 0.52° was retrieved. For this specific case, the maximum absolute error was found to be 1.73°. Finally, concerning the seated knee flexion, the mean absolute error provided by the automatic measurement was equal to 2.23°, with a variance of 1.43° and a maximum error of 3.21°. These values were considered sufficiently accurate for a home-based measurement by a team of physiotherapists working at the Villa Olivella Hospital located in Florence (Italy). Furthermore, these results are comparable both to the ones obtained by using 3D-scanner based measurements, such as the one proposed in [19] where the relative measurement of knee internal/external rotation (mean (SD)) offset error was 3.4 degrees with a mean RMS error of 1.6°, and to those of IMU-based systems, which have an average error equal to ±3° [20].

From the preliminary results obtained during experimentation, the proposed system allows for complete monitoring of not only the standing output, as recorded in previous similar systems [8], but also of supine exercises, as in the case of actual post-operative rehabilitation. The recorded joint angles show a correct representation of both the exercise repetitions and the corresponding joint angle reached each time, even in the presence of substantial noise signals. The surgeon and/or physiotherapists, to determine whether the patient completes a successful recovery at home or whether any clinicians need to correct or assist him, may review such graphs.

## 4. Discussion

The main aim of the present work was to provide a two-step interactive approach for the real-time monitoring of patients during their home treatment in order to address the key drawback of using a pure 2D or 3D tracking algorithm. The method, which employs both 2D and 3D acquisition devices, is a hybrid and interactive approach for the real-time measurement of lower limb joint rotation angles during post-TKA and -THA surgery recovery. In the proposed method, a 2D acquisition method was used to estimate the patient’s 2D body posture. Subsequently, the depth information coming from the RGB-D sensor was used to determine the spatial range of a patient’s lower joints so as to calculate the 3D positions of the joints.

Consequently, it is possible to identify the joints of a human body in a complex environment in almost all the possible poses (i.e., including non-frontal poses) even though the output is purely the pixel positions of the joints.

By leveraging depth information from an RGB-D sensor, the developed method can measure the 3D locations of each lower joint (hip and knees) returned by the 2D tracker until the body posture is recovered.

The preliminary results obtained showed that the proposed method effectively overcomes many limitations by fusing the results obtained by the state-of-the-art robust 2D pose estimation algorithms with the 3D data of depth cameras by allowing the patient to be tracked in unregulated positions, thus not being limiting to just standing exercises. In fact, for the preliminary assessment of the method, the mean error obtained for several repeated tests is in the range of ±3°, which can be considered sufficiently accurate for a system to be operated autonomously by a patient outside the hospital structure.

The devised system is intended to be used by the patient in a home environment, thus allowing for the tracking of the correctness of the performed rehabilitation exercises. In fact, the devised system can be easily deployed at home, since it consists of low-cost devices that the hospital could offer to the patient for rehabilitation purposes. By using such a device, the angular extent of the patient’s lower joints may be calculated, and this detail may be used (by doctors) to ascertain the correctness of home-based therapy.

With this system, the surgeon and/or physiotherapists may determine whether the patient completes a satisfying recovery at home or whether she/he needs to be further assisted by a doctor by receiving real-time information from the patient, as mentioned in [20]. Therefore, in the near future, a method for the real-time transmission of data from the acquisition system and a server is envisaged. Stored data will be shared with the doctors in order to track the progress of the patient during rehabilitation. In addition, the self-use of the proposed interactive technology can enable patients (especially younger ones) to perform the required rehabilitation exercises more assiduously and correctly [21].

It has to be considered that according to recent review studies [22,23], 2D and 3D devices will be increasingly used for patient care, even if a better understanding of the facilitators and barriers to the feasibility of using this kind of technology in a real-world setting is recommended. Technical complexities, price, data quality concerns and unclear end-user needs are, in fact, some of the barriers to the wide spreading of these systems [24]. For this reason, the proposed system aims to reach a low complexity level of interaction with the patient together with the use of low-cost devices. In fact, for home-based use, the system should be accompanied with a set of clear instructions to guide the patient towards correct use and should be simplified in terms of user interface [25].

It has to be considered that the proposed system has a number of limitations, and further research should be performed in the near future. In fact, results show that the accuracy of the system is highly influenced by the body parts’ possible optical occlusion as well as by the offset of the skin from the real articulation joints. Moreover, the system is able to provide only a rough estimation of the angles of rotation, even if the obtained accuracy is sufficient to monitor the movements in the specific physiotherapy exercises. Future works will be aimed towards performing more validation tests on a larger number of users to derive other possible drawbacks of the proposed method and to draft possible improvements. Moreover, the system should be improved in terms of usability by devising user-friendly GUIs. Finally, an information interchange system should be devised to share the home-based exercises with the physicians to allow for a continuous monitoring of the patients’ progress.

## 5. Conclusions

Home-based rehabilitation is becoming the gold standard for patient wellness because it helps to reduce healthcare costs. However, there is a risk of worsening adverse health effects in the case of home care, owing to the patients’ lack of motivation and the doctors’ difficulty in providing rigorous supervision. This is particularly true when a patient has gone through knee arthroplasty or full knee replacement. In these cases, correct supervision can be considered a crucial issue, since recovery requires following a customized treatment protocol that is often burdensome for the patient. As a result, the creation of devices to measure the efficient recovery of the operated joint is highly valued both for the patient, who will feel encouraged to complete the appropriate number of activities, and for the doctor, who will be able to track him/her remotely. This should be performed by means of low-cost devices and simple methods, which allows a correct measurement of a range of angle of interest but without requiring complex installations. Consequently, in the present work, a method based on two steps was developed. The proposed method proved to overcome the main limitation of using a pure 2D or 3D skeleton tracking algorithm for monitoring patients in home care.

## Figures and Tables

**Table 1:** Recommended remote exercises for the first 7 days of post-operative TKA.

TKA Exercise	Reps	Rest Interval
Isometric quadriceps contraction, 8 s	15	60 s
Leg raise, 9 s	15	120 s
Supine knee flexion, 12 s	15	180 s
Seated knee flexion, 12 s	15	180 s
High knees, 9 s	15	

**Table 2:** Recommended remote exercises for the first 7 days of post-operative THA.

THA Exercise	Reps	Rest Interval
Hip bridge, 12 section	15	180 section
Supine hip flexor, 12 section	15	60 section
Butterfly stretch, 12 section	15	180 section
Seated leg extension, 7 section	15	120 section
Standing hip abduction, 8 section	15	
